# Effectiveness of mesenchymal stem cells cultured under hypoxia to increase the fertility rate in rats (*Rattus norvegicus*)

**DOI:** 10.14202/vetworld.2021.3056-3064

**Published:** 2021-11-30

**Authors:** Erma Safitri, Hery Purnobasuki

**Affiliations:** 1 Department of Veterinary Science, Division of Veterinary Reproduction, Faculty of Veterinary Medicine, Universitas Airlangga, Surabaya 60115, Indonesia; 2 Department of Biology, Faculty of Science and Technology, Universitas Airlangga, Surabaya 60115, Indonesia

**Keywords:** fertility, hypoxia culture, malnutrition, stem cell, testicular failure

## Abstract

**Background and Aim:**

Mesenchymal stem cells (MSCs) transplanted into the testes of rats with testicular failure can help rescue fertility. However, the low viability of transplanted MSCs limits the success of this treatment. This study aimed to determine the effectiveness of MSCs cultured under hypoxia to increase the fertility rate in rats (*Rattus norvegicus*).

**Materials and Methods:**

Bone marrow-derived MSCs (200 million cells/rat) were transplanted into male rat models with induced infertility (10 rats/treatment group) after 4 days of culture in 21% O_2_ (normoxia) and 1% O_2_ (hypoxia). Ten fertile and 10 untreated infertile rats served as controls. In the infertile male rats that had been fasted from food for 5 days, the fasting condition induced malnutrition and then resulted in testicular failure.

**Results:**

The results indicated that the MSCs cultured under hypoxic conditions were more effective than those cultured in normoxic conditions as a treatment for testicular failure in infertile male rats based on the increased number of cells expressing p63 as a quiescent cell marker and ETV5 as a transcription factor expressed in Sertoli and germ cells. Furthermore, the structure of the seminiferous tubules, which contain spermatogonia, primary and secondary spermatocytes, and spermatid, Sertoli, and Leydig cells, was improved in infertile male rats treated with the MSCs cultured under hypoxic conditions.

**Conclusion:**

The testicular transplantation of MSCs cultured under hypoxic conditions was an effective treatment for testicular failure in rats.

## Introduction

Mesenchymal stem cell (MSC) transplantation using rabbit [1] and rat [2] bone marrow and later rat [3] and rabbit [4] adipose tissue was shown to be effective in rebuilding the tissues supporting the endogenous stem cells, allowing them to multiply and mature into sperm cells. The viability of the transplanted MSCs from the bone marrow [2,5], adipose tissue [3], or umbilical cord blood [6] is low; hence, this treatment has limited efficacy. The reduced viability of MSCs is thought to be caused by a normoxia culture with a high oxygen concentration (O_2_>20%). Cell senescence [7], cell apoptosis [8], and gene mutation [9] can all be caused by normoxia culture. As a result, the low viability of MSCs restricts the success of cell transplant therapy. It is hypothesized that the efficiency of MSC transplantation is influenced by apoptosis [10-15]. To achieve therapeutic impact, substantial doses of MSCs are necessary. Several researchers are attempting to acquire adequate dosage without the use of boosters, hence reducing the influence on rising expenses. Due to these issues, the effectiveness of the treatment remains unclear. Thus, alternative investigations are still required to learn more about the efficacy of more relevant treatments.

Other studies have indicated the critical significance of stem cell cultivation under hypoxic conditions as follows: To keep transplanted MSCs alive and adaptable, they were grown in a hypoxic environment (1-3% O_2_) [2,4]. This condition was induced by cells in the quiescent state [4,16,17], allowing the cells to live longer [18,19]. Hypoxia-inducible factor 2 (HIF2), a critical regulator of progenitor stem cell function, may influence the expression of p63 as a definite marker in quiescent cells. Quiescent MSCs are self-renewing stem cells that remain in gap 0 and do not cycle (i.e., gap 1/synthesis/gap 2/mitosis) [20] or in undifferentiated states [21]. However, there is still a high potential for cell renewal [22]. At the indifferent stem stage, self-renewal is a symptom of the biological process and defense mechanism [23]. The homing signal based on the vascular endothelial growth factor (VEGF) expression following transplantation is critical for culture hypoxia-conditioned rat MSCs (1% O_2_ concentration) [2]. VEGF is a component of the stem cell extracellular matrix that helps maintain a favorable milieu for stem cells to survive after transplantation.

Scientific evidence on the effectiveness of MSCs cultured under hypoxia for testicular failure is still lacking. Therefore, we determined whether HIF2 (with HIF2 alpha [HIF2α] monoclonal antibody [ep190b] as a marker) regulated the transplantation of MSCs in the form of quiescent MSCs (with p63/TP73L monoclonal antibody as a marker) derived from rat bone marrow and whether HIF2 played a role crucial for spermatogonial stem cells (SSCs). Infertile males with testis failure can be treated with transplantation of MSCs cultured under hypoxic conditions. The findings of this study are relevant in the area of male reproductive health.

This study aimed to determine the effectiveness of MSCs cultured under hypoxia to increase the fertility rate in rats (*Rattus norvegicus*).

## Materials and Methods

### Ethical approval

The study was approved by Animal Care and Use Committee (No: 239-KE; *Komisi Etik Penelitian*) of the Faculty of Veterinary Medicine, Universitas Airlangga, Surabaya, Indonesia.

### Study period and location

The study was conducted from March 2018 to 2020 at Department of Veterinary Science, Faculty of Veterinary Medicine, Universitas Airlangga, Surabaya, East Java, Indonesia.

### Stem cell isolation

Stem cells were harvested from the bone marrow through aspiration of the femur, tibia, and ulna [24] of 3-month-old rats (*Rattus norvegicus*) [25]. The aspirate was placed in heparinized tubes (Z181099, Sigma-Aldrich^®^, Burlington, Massachusetts, USA) and stored at 4°C to be transported to the laboratory [26].

### Stem cell culture

The aspirate from the rat bone marrow was transferred into 15 mL sterile tubes (SIAL0790-500EA, Sigma centrifuge tubes, Sigma-Aldrich^®^), rinsed twice with 5 mL sterile phosphate-buffered saline (PBS), (MFCD00131855, Sigma-Aldrich^®^), and filled up to a total volume of 10 mL. The diluted sample was added with the same volume of Ficoll (Biowest, Nuaillé, France) in a separate 15 mL tube. Centrifugation was performed for 15 min at room temperature (37°C) at 1600 rpm. After centrifugation, the cells were collected from the Ficoll-PBS interface using a sterile Pasteur pipette (Corning™ C7095BNMR, Thermo Fisher Scientific, Waltham, MA, USA) and transferred into a 15 mL tube. The cells were resuspended in PBS up to a total volume of 15 mL. The tube was gently inverted and shaken (CLS6791 Sigma, Corning LSE Benchtop Shaking Incubator with Platform, Sigma-Aldrich^®^) 5 times to homogenize the suspension.

The suspension was centrifuged again for 10 min. The supernatant and floating cells were discarded, and the cell pellet was resuspended in 6 mL of alpha-modified essential medium (α-MEM) (M0894; Sigma-Aldrich). Mononucleated cells were placed on a plate in 10 cm^2^ with approximately 2×10^7^ cells and incubated at 37°C in a humidified atmosphere with 5% CO_2_ (BioSpherix, Florida, USA) for 24 h to let the cells adhere (sticking in a plate). After 24 h, the media and non-adherent cells were discarded. The adherent cells were rinsed twice with 5 mL PBS, and 10 mL fresh α-MEM medium was then added into the dish, which was returned into the incubator. The culture was observed daily under an inverted microscope. The medium was changed every 4 days, preceded by a rinse with 10 mL PBS, after which 10 mL fresh α-MEM medium was added. The culture was continued until approximately 75-80% confluence was achieved. After confluence, the cells were passaged into several other dishes to cultivate subcultures [26]. Passaging was performed three times, and then, the cells were assigned to two conditions: Hypoxic precondition treatments of 1% in a hypoxia chamber (BioSpherix, Florida, USA) inside a 5% CO_2_ incubator and another treatment using 21% O_2_ concentration (normoxia) over 4 days. The MSCs were observed under a microscope before being transferred into the testes.

### Infertility rat model

This study used male rats with testicular failure that had been fasted from food for 5 days but provided with drinking water *ad libitum* [2,27]. The fasting condition for 5 days induced malnutrition and then resulted in testicular failure. The malnutrition condition caused the adrenal cortex to function suboptimally in producing dehydroepiandrosterone (DHEA). Low levels of DHEA in the blood can cause fatigue and decrease sperm concentration. DHEA is the most potent precursor of steroid hormones, such as testosterone, which is produced by the renal adrenal cortex [28] and Leydig cells of the testis [29]. Low testosterone production can lead to decrease spermatogenesis and, thus, testicular failure. The animal models used in this study were healthy 8-10-week-old Wistar strain male rats (*R. norvegicus*) with a body weight of 250-300 g. The rats were placed in individual plastic cages in the experimental animal laboratory at the Faculty of Veterinary Medicine, Universitas Airlangga.

### MSC transplantation methods

The MSCs were transplanted into male rats with testicular failure, which were then compared with the negative and positive control rats. The T1 group consisted of 10 infertile male rats that were transplanted with stem cells cultured under normoxia (21% O_2_ concentration) for 4 days with a dose of 200 million cells/rat [1]. The T2 group consisted of 10 infertile male rats that were transplanted with stem cells cultured under hypoxia (1% O_2_ concentration) for 4 days with a dose of 200 million cells/rat [1]. The positive (fertile) control group was composed of 10 normal male rats (fertile) injected with 0.1 mL PBS. The negative (infertile) control group consisted of 10 infertile male rats injected with 0.1 mL PBS.

The testes of the male rats were surgically excised after 54 days (one cycle of spermatogenesis) [30] to collect testicular tissue. The improvement in the testicular tissue was observed by histopathological preparations using hematoxylin and eosin (H&E) stain (B8438, Sigma-Aldrich^®^). Immunohistochemical (IHC) observation was performed to determine the expression of p63 (with p63/TP73L monoclonal antibody) as a marker of quiescent cells, HIF2α (with HIF2α monoclonal antibody [ep190b]) as a marker that is crucial for endogenous cells (SSCs, Sertoli cells, etc.), and ETV5 (with ETV5 monoclonal antibody) as a marker for the transcriptional factor to improve testicular failure and infertility. *In vitro* fertilization between the ovum and sperm cells was performed to observe the fertility of the male rats.

### Histopathological assessment

Histopathological examination of the testicular tissues for the presence of Sertoli cells, Leydig cells, spermatogonia, spermatocytes, and primary and secondary and spermatid cells was performed on the testicular tissues that were fixed with 10% formalin. The testes were then dehydrated through a series of increasing alcohol concentrations, cleared with xylol, and embedded in paraffin. Thin sections mounted on slides were processed for H&E staining [31].

Histopathological examination was performed using a light microscope with a magnification of 200×. Five fields of view were assessed for each slide. Observations and identification of the spermatogonia and Sertoli and Leydig cells and regeneration of seminiferous tubules were based on the existing histological description [27].

### IHC observation

IHC observation was performed to determine the expressions of p63, HIF2α, and ETV5. The samples were prepared for histopathological examination of the testicular tissue. After deparaffinization of the preparation (paraffin block) with xylene 3 times each for 3 min, rehydration of the preparation with 100%, 95%, and 70% ethanol each for 2, 2, and 1 min and finally with water for 1 min was performed. Subsequently, the preparation was soaked in peroxidase blocking solution at 37°C for 10 min and then incubated in pre-diluted blocking serum at 25°C for 10 min.

In the next procedure, the IHC technique used monoclonal antibodies to determine the expressions of p63, HIF2α, and ETV5. The preparation was soaked in p63 (MAB1916, Monoclonal Anti-p63/TP73L antibody, R&D Systems Inc., Minneapolis, USA), HIF2α, (MA1-16519, HIF-2α Monoclonal Antibody (ep190b), Thermo Fisher Scientific), and ETV5 (MBS831454, Mouse anti-Human ETV5 Monoclonal Antibody, MyBioSource, San Diego, USA) monoclonal antibodies for 10 min and then washed with PBS for 5 min.

Subsequently, the preparation was incubated with a secondary antibody (conjugated to horseradish peroxidase) at 25°C for 10 min, washed with PBS for 5 min, and then incubated again with peroxidase at 25°C for 10 min. Next, the preparation was washed with PBS for 5 min and then incubated with chromogen diaminobenzidine at 25°C for 10 min.

Furthermore, the preparation was incubated with H&E for 3 min and washed with water. Finally, the preparation was cleaned, dropped with mounting media, and closed with a coverslip. Then, the expressions of p63, HIF2α, and ETV5 (brown color) were observed on the cells using a light microscope with 400×. Five fields of view (one tubule/field view) were assessed for each slide through the scoring system. The following IHC scoring system [32] was used: IHC score = A×B, where A denotes the wide percentage of expressions and B is the intensity of the chromogen color (Table-1).

**Table-1:** Semi-quantitative IHC scale taking into account both percentage of positive cells (A) and intensity of reaction color (B) with the final score representing product of the two variables (A×B).

A	B
0 patients no cells with positive reaction	0 patients no color reaction
1 patient 10% cells with positive reaction	1 patient low intensity of color reaction
2 patients 11-50% cells with positive reaction	2 patients moderate intensity of color reaction
3 patients 51-80% cells with positive reaction	3 patients intense color reaction
4 patients>80% cells with positive reaction	

IHC=Immunohistochemical

### Medium preparation, sperm and oocyte collection, and *in vitro* fertilization

The media, M16 (MR-016 EMD Millipore, Sigma-Aldrich Inc., Darmstadt, Germany) and PBS (10010031 PBS, pH 7.4; Thermo Fisher Scientific), were manufactured in accordance with the established procedure for producing these two media. Before use for *in vitro* fertilization, a droplet medium was prepared in a Petri dish (Sterilin™ 100 mm Petri Dishes, Thermo Fisher Scientific) with a volume of 50 μL as a washing medium and 25 μL as an *in vitro* culture medium. The droplet medium was then incubated for 3 h in a 5% CO_2_ incubator at 37°C before being used for *in vitro* fertilization [33].

Sperm cells were collected from male rats after being sacrificed by dislocation of the fourth cervical spine and then disinfected with 70% alcohol. A Y-shaped incision was made in the abdomen, the stomach contents were removed, and the left testicle was pulled out. The fats were separated, and then part of the cauda epididymis, the mature sperm storage, was taken. The obtained cauda epididymis was washed with PBS twice, cut into small pieces to free the spermatozoa, placed on M16, and then incubated in a 5% CO_2_ incubator at 37°C [34].

Before the oocytes were collected, PMSG and hCG hormones were injected subcutaneously to stimulate superovulation: On the 0^th^ h on the 1^st^ day, female rats were injected with 5 IU of 0.1 mL PMSG to stimulate the process of folliculogenesis and left to stand for 48 h. The female rats were injected with 5 IU of 0.1 mL hCG 48 h after the PMSG was injected and mated directly with a single vasectomized male to stimulate ovulation. After 17 h, a vaginal plug was used to confirm that the female had mated. Then, the oocytes were flushed. The female rats were killed by dislocation of the fourth cervical spine. A Y-shaped incision was then made on the abdomen, and the uterus was removed and separated from the fallopian tube section and then rinsed with M16. The oocytes from the ampulla of the fallopian tube were flushed using an inverted microscope [35].

### Fertility rate observations

The fertility of spermatozoa was determined by examining *in vitro* fertilization. Semen analysis was conducted according to the guidelines of the World Health Organization. Semen was processed over a two-layer discontinuous density gradient, formed by a top layer of 40% (v/v) PureSperm (Nidacon Lab AB, Gothenburg, Sweden) and a lower layer of 80% (v/v) PureSperm, by centrifugation at 1500 g for 15 min at 37°C. The pellet was resuspended in 3 μL SAGE fertilization medium with 5% HSA and spun down at 200 g for 10 min at 37°C [36]. The oocyte and sperm were both placed in the Petri dishes containing M16 drops under a mineral oil overlay and incubated in 5% CO_2_ incubators at 37°C for 5 h for *in vitro* fertilization [37]. Fertilization was confirmed by the presence of the second polar body of oocyte [36] through an inverted microscope (Nikon Eclipse TE 2000S; Nikon, Tokyo, Japan) at 400× with Hoffman modulation optics. In rats, the first polar body is known to degenerate.

The embryo quality was evaluated on days 2-3. On days 2-3, embryo development was assessed, including blastomere number, size, regularity, presence, and percentage of fragmentation. All embryos were graded on a scale of 1-5, with 1 being the best. Grade 1 embryos had symmetrical blastomeres of equal size with no cytoplasmic fragmentation. Grade 2 embryos had blastomeres of equal size and minor cytoplasmic fragmentation covering <10% of the embryo surface. Grade 3 embryos had even or uneven blastomeres and minor cytoplasmic fragmentation covering 10-25% of the embryo surface. Grade 4 embryos had blastomeres of equal or unequal size and moderate to high cytoplasmic fragmentation covering 25-50% of the embryo surface. Grade 5 embryos contained few blastomeres of any size and severe fragmentation covering >50% of the volume of the embryo. Embryos with good quality were defined as those with six to eight equal-sized cells and <10% fragmentation on day 3 [36]. The fertility rate of sperm formula in rats was calculated as follows: Number of good-quality embryos/the number of mature oocytes × 100% [35].

### Statistical analysis

The expressions of p63, HIF2α, and ETV5 and the fertility rate of the sperm cells were statistically analyzed using the SPSS software (v. 17 for Windows XP; SPSS Inc., Chicago, IL, USA) with a 99% confidence level (α=0.01) and 0.05 significant difference (p<0.05). The comparative steps for hypothesis testing were as follows: Normality data test, Kolmogorov-Smirnov test, homogeneity of variance test, analyses of variance, and Tukey’s HSD *post hoc* test with 5% least significant difference.

## Results

Data were collected from 40 male rats, which were divided into four treatment groups: Normal males in the positive (fertile) control, infertile males treated with PBS in the negative (infertile) control, infertile males transplanted with stem cells cultured under normoxia (21% O_2_ concentration) for 4 days in the first treatment (T1) group, and infertile males transplanted with stem cells cultured under hypoxia (1% O_2_ concentration) for 4 days in the second treatment (T2) group. The results indicated that transplantation with MSCs from hypoxic precondition culture improves testicular function by decreasing the extent of damage and increasing fertility. The expression levels of p63, HIF2α, and ETV5 increased, as were the regeneration of the testicular tissue, as described in more detail in the following, such as intact tubular seminiferous tissue; formation of Sertoli cells, Leydig cells, spermatogonia, spermatocytes, and primary-secondary and spermatid cells; and improvement of the fertility rate of sperm *in vitro*.

### Expression of p63

**Figure-1:**
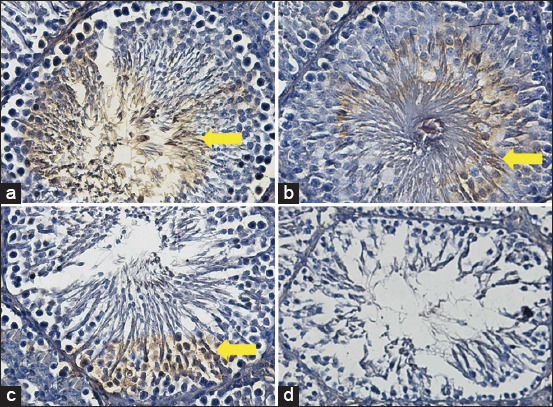
Average score of p63 expression (brown): (a) Positive (fertile) control group=9.6^a^±0.44; (b) T2 group=6.0^b^±0.50; (c) T1 group=2.5^c^±0.25; (d) negative (infertile) control group=0.41^d^±0.22. (a-d) 400× with the IHC method. IHC=Immunohistochemical.

The average score of the p63 expression in the T2 group was 6.0^b^±0.50, although the score was lower than that in the positive (fertile) control group, in which p63 was expressed (9.60^a^±0.44), but the score was still much higher than that in the T1 (2.5^c^±0.25) and negative (infertile) control groups, in which p63 was not expressed (0.41^d^±0.22) (Figure-1 and Table-2).

**Table-2:** The average score of p63, HIF2α, and ETV5 expression in some treatments as shown by the IHC method in testicular tissue of rat.

Treatments	Sample (n)	Score±SD		

		Average score p63 expression	Average score HIF2 a expression	Average score ETV5 expression
Positive (fertile) control group	10	9.6^a^±0.44	0.6^c^±0.34	10.5^a^±0.25
Negative (infertile) control group	10	0.41^d^±0.22	0.3^c^±0.33	0.3^d^±0.23
Infertile male transplanted with stem cells from normoxia culture (21% O2 concentration) (T1 group)	10	2.5^c^±0.25	3.9^b^±0.44	2.2^c^±0.15
Infertile male transplanted with stem cells from hypoxia culture (1% O_2_ concentration) (T2 group)	10	6.0^b^±0.50	7.0^a^±0.75	7.2^b^±0.34

^a-d^Different superscripts in the same column were significantly different (p<0.005). IHC=Immunohistochemical, HIF=Hypoxia-inducible factor

### Expression of HIF2α

**Figure-2:**
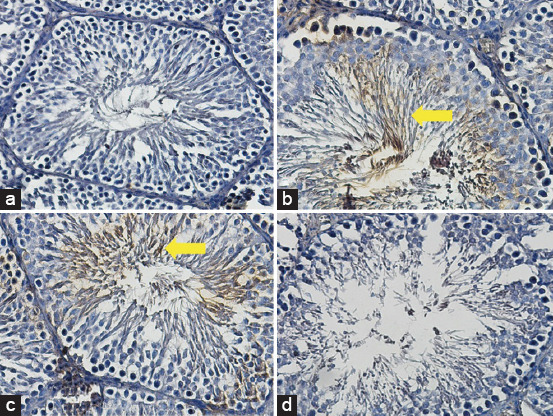
Average score of HI2α expression (brown): (a) Positive (fertile) control group=0.6^c^±0.34; (b) T2 group=7.0^a^±0.75; (c) T1 group=3.9^b^±0.44; (d) negative (infertile) control group=0.3^c^±0.33. (a-d) 400× with the IHC method. IHC=Immunohistochemical, HIF=Hypoxia-inducible factor.

The average score of the HIF2α expression in the T2 group was 7.0^a^±0.75. This was the highest among those in the other groups, T1 (3.9^b^±0.44), positive (fertile) control (0.6^c^±0.34), and negative (infertile) control (0.3^c^±0.33) (Figure-2 and Table-2). An increase in the score of the HIF2α expression could occur if they were given a low O_2_ concentration.

### Expression of ETV5

**Figure-3:**
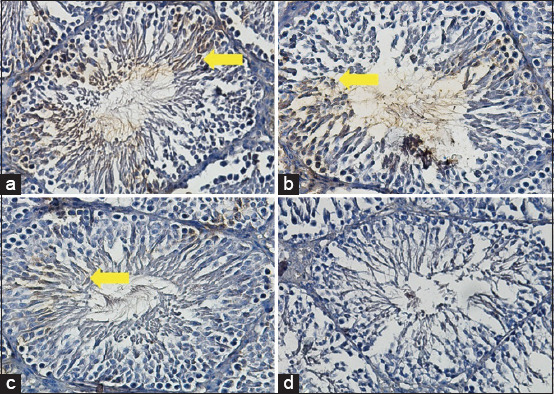
Average score of ETV5 expression (brown): (a) Positive (fertile) control group=10.5^a^±0.25; (b) T2 group=7.2^b^±0.34; (c) T1 group=2.2^c^±0.15; (d) negative (infertile) control group=0.3^d^±0.23. (a-d) 400× with the IHC method. IHC=Immunohistochemical.

The average score of the ETV5 expression in the T2 group was 7.2^b^±0.34, although the score was lower in the positive (fertile) control group (10.5^a^±0.25), but the score was still higher than that in the T1 (2.2^c^±0.15) and negative (infertile) control groups, in which ETV5 was not expressed (0.3^d^±0.23) (Figure-3 and Table-2).

### Regeneration of testicular tissue

**Figure-4:**
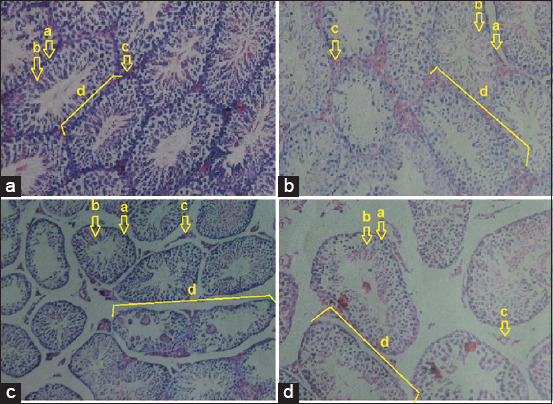
Rat testicular tissue with hematoxylin and eosin (H&E) staining: (A) Positive (fertile) control group: (a) spermatogonia cells, (b) Sertoli cells, (c) Leydig cells, and d. intact tubules seminiferous; (B) T2 group: (a) Spermatogonia cells, (b) Sertoli cells, (c) Leydig cells, and (d) intact tubules seminiferous; (C) T1 group: (a) Spermatogonia cells, (b) Sertoli cells, (c) Leydig cells, and (d) damaged tubules seminiferous; (D) negative (infertile) control group: Loss of (a) spermatogonia cells, (b) Sertoli cells, (c) Leydig cells, and (d) damaged tubules seminiferous. (A-D) 200×. H&E: Hematoxylin and eosin.

Microscopic examinations of five different fields of view revealed that the T2 group experienced repaired testicular tissue. The improvements were identified based on the regeneration of Sertoli cells, Leydig cells, spermatogonia, spermatocytes, primary-secondary and spermatid cells, and seminiferous tubules. An overview of these improvements could be compared with the positive (fertile) control, which did not experience testicular degeneration. The T2 group remained under the normal condition (Figures-4a and d, Table-3). The T1 group did not exhibit testicular tissue repair, indicating that intact seminiferous tubules were not observed, and spermatogonia, Sertoli cells, and Leydig cells were degenerated. This amount of damage was comparable to that of the negative (infertile) control (Figures-4b and c, Table-3).

**Table-3:** The average number of Sertoli cells, Leydig cells, spermatogonium, spermatocyte primary-secondary, and spermatid cells in some treatments.

Treatments	Sample (n)	Average number of Sertoli cells±SD	Average number of Leydig cells±SD	Average number of spermatogonium cells±SD	Average number of spermatocyte primary-secondary cells±SD	Average number of spermatid cells±SD
Positive (fertile) control group	10	11.5^d^±0.45	29.56^d^±0.85	30.65^d^±0.70	74.25^d^±1.65	85.55^d^±1.50
Negative (infertile) control group	10	2.1^a^±0.47	5.15^a^±0.75	5.35^a^±0.75	30.15^a^±1.70	12.25^a^±1.75
Infertile male transplanted with stem cells from normoxia culture (21% O_2_ concentration) (T1 group)	10	4.2^b^±0.30	10.35^b^±0.65	12.95^b^±0.60	43.45^b^±1.45	30.50^b^±1.30
Infertile male transplanted with stem cells from hypoxia culture (1% O_2_ concentration) (T2 group)	10	7.8^c^±0.53	17.85^c^±0.75	20.75^c^±0.80	69.85^cd^±1.50	55.25^c^±1.25

^a-d^Different superscripts in the same column were significantly different (p<0.005)

The number of different types of cells was counted based on the characteristics of each cell, as in the following:

Spermatogonium: It has a round shape and is located near the basement membrane, and the nucleus has an oval shape with fine chromatin and a thin nuclear membrane.

Primary spermatocyte: It has the largest size among gamete cells, with heterochromatin in the nucleus, and is located between the basal membrane and the tubular lumen.

Secondary spermatocyte: It is rarely observed in the seminiferous tubules because it quickly divides into spermatids.

Spermatid: It has a round shape and is smaller than spermatocytes, and the nucleus is round, pale, and bright.

Sertoli cell: It has a slim, irregular shape, and the base attaches to the basement membrane of the seminiferous tubules, having one nucleus located at the center.

Leydig/interstitial cell: It is located in the loose connective tissue between the tubules; this is a large cell, polygonal in shape, and the nucleus is clearly visible and also polygonal in shape.

### Improvement of the fertility rate of the sperm

The T2 group exhibited a significant improvement in the fertility rates compared with the T1 group, although the rate did not reach that of the positive (fertile) control group (Table-4).

**Table-4:** Fertility rate of sperm of male rat.

S. No.	Treatment	Number of mature oocyte	Good quality embryos	Fertility rate (%)
1.	Positive (fertile) control group	50	43	86
2.	Negative (infertile) control group	50	2	4
3.	Infertile male transplanted with stem cells from normoxia culture (21% O_2_ concentration) (T1 group)	50	9	18
4.	Infertile male transplanted with stem cells from hypoxia culture (1% O_2_ concentration) (T2 group)	50	31	62

## Discussion

In this study, we determined whether MSCs exposed to hypoxic conditions could repair testicular function more effectively than MSC cultured under normoxic conditions. Hypoxia, in this study, was adjusted to the normal and physiological conditions in which the stem cells were *in situ* (*in vivo*). Therefore, an *ex situ* (*in vitro*) study was conducted by inducing hypoxic conditions so that the conditions of the stem cells cultured are the same as the *in situ* physiological conditions (*in vivo*). In *in vivo* conditions, the MSCs are in the form of quiescent.

The induction of quiescent MSCs through p63 quiescent expression increased the life expectancy of stem cells by maintaining their viability and the adaptive condition for stem cell transplantation. The role of hypoxia in maintaining quiescence stem cells begins with the induction of HIF2α. Subsequently, HIF2α activated the gene pluripotency after it had been preceded by an initial adaptation time by HIF1α. The pluripotency of these stem cells may prolong the lifetime of quiescent cells so that the function of stem cells (stemness) is maintained. Furthermore, after transplantation, it mobilized the endogenous stem cells toward the defect area (testicular tissue). The process of mobilization can occur through several ways: (a) Induction of proteolysis (protein degradation) from the microenvironment of bone marrow, such as induction of pharmacological agents (granulocyte-colony stimulating factor or cyclophosphamide) or induction of quiescent stem cells that were transplanted (p63 marker); (b) blockade of CXCR4 or VLA-4 by specific blocking molecules, such as AMD3100 or BIO4860; (c) effect from neural mediators, such as dopamine and beta-2 adrenergic receptors; (d) element modulation from the coagulation of cascade apoptosis; (e) inflammatory reaction causing injury signals or immune response induction, such as NF-κB, cytokines, or β-catenin, through Wnt from the tissue damage; and (f) homing signals, such as SDF1, CXCL12, VEGF, HGF, PDGF, and integrin, or transcriptional factors, such as HIF2α and ETV5, which appeared and recruited endogenous stem cells [27]. The mobilization of endogenous stem cells will further move the stem cells to the testicular tissue, resulting in spermatogenesis processes and rescue of testicular failure and infertility repair.

This study demonstrated that the MSCs from the hypoxic precondition culture were effective for therapy in male rats with testicular failure and infertility based on the increased of p63 expression as a quiescent cell marker as a crucial for progenitor of stem cell function and ETV5 expression as a transcriptional factor for regeneration of testicular tissue and improvement of the fertility of sperm.

The regenerative efforts of stem cells were differentiated by the decreased expression of p63 compared with that in the positive (fertile) control group. This study demonstrated that the score of p63 in the T2 group was lower than that in the positive (fertile) control group but was still better than those in the T1 and negative (infertile) control groups, which is slightly expressed (Figure-1 and Table-1).

The p63 gene can maintain the viability of stem cells and regenerate stem cells from various tissue cells, which are known as ringmasters. In the previous study [4], the absence of p63 was showed a decreased of proliferation ability of cells, indicating that p63 is a key function in increasing the division of stem cells because the p63 gene directly promotes and control the stem cell environment and maintain undifferentiation.

Hypoxic precondition led to HIF1α release; therefore, so that not bound by the van Hippel-Lindau factor as a factor inhibited for HIF1α action furthermore[38]. Furthermore, HIF1α would be bound to HIF1β so that complex HIF1 was formed [9]. The HIF1α+HIF1β bond occurred in specific DNA sequences known as the hypoxia response element (HRE) 5’-TACGC-3’. Complex HIF1α and HIF1β bonds on HRE occurred at the start of exposure to hypoxia [39], thus causing cell cycle arrest and gene expression [40]. This inhibited the p21 expression, resulting in cell cycle inactivation andresistance to senescence and exhausting cells [7]. This is thought to slow down the proliferation of cultured stem cells; thus, quiescent cells can still be maintained [41].

Long-term maintenance of quiescent cells was also thought to be influenced by cultivation time-dependent hypoxic preconditions. After 48 h under low O_2_ tension, the role of HIF1α would be replaced by that of HIF2α with different target genes [39]. The target genes in *in vitro* culture were expected to induce the expression of pluripotency genes [42], such as OCT4, SOX2, NANOG [9,43], and REX-1 [23]. A hypoxic precondition is an effort to change the ability of multipotent stem cells to become pluripotent.

The mean identification of the HIF2α score in the T2 group was the highest among those of the other groups. *In vitro* cultures, low oxygen tension (hypoxia), and cultivation time-dependent administration of oxygen induce expressions of pluripotency genes [37], such as OCT4, SOX2 [1,44], REX-1 [44], and NANOG [45]. Pluripotency genes are activated by HIF2α [46] after preceding initial adaptation time by HIF1α [9]. The pluripotency of these stem cells can retain quiescent cells; therefore, the function of stem cells is maintained. The quiescent cells with p63 as a marker, which is regulated by HIF2α from hypoxic precondition culture with 1% O_2_ concentration for 4 days, are crucial for conducive niche *in vivo*, so that after transplantation, the stem cells could be transdifferentiated through spermatogenesis in the seminiferous tubules of the testis.

Furthermore, stem cells from the hypoxic precondition culture were found to be effective based on ETV5 formation in the testicular tissue, with an average score of 2.95^b^±0.50. The ETV5 as a marker of SSC function that can increase and improve the testicular environment and support endogenous stem cells, so that stem cells can be mobilized to the testicular tissue that has failed, resulting in improvement and rescue for fertility.

In this study, IHC methods were employed to identify ETV5. The score of the ETV5 expression in the T2 group was approximately 3. Although this score was below that of the positive (fertile) control group, it was still well above those of the T1 and negative (infertile) control groups, which was slightly expressed (Figure-3 and Table-1). Previous research demonstrated that bone marrow-derived MSCs are adult stem cells that quickly grow and differentiate into cells that are needed in response to the presence of defects [40].

Regeneration of the testicular tissue was identified as an intact seminiferous tubule tissue; formation of Sertoli cells, Leydig cells, spermatogonia, spermatocytes, and primary and secondary and spermatid cells. The viability of stem cells that differentiate into cells is necessary. In infertile conditions, the degenerative testicular tissue can be regenerated if the stem cells are viable. If they are not viable, then the testicular tissue will remain degenerated.

The survival of stem cells in the animal model of degenerative tissue, such as testicular failure, is beyond the scope of the therapeutic effect of MSC treatment. In addition, poor survival following cell transplantation is a crucial factor [5]. This study demonstrated that stem cells from the hypoxic precondition culture survived based on the effectiveness of therapy in male rats with testicular failure and infertility through the regeneration of their testes, which can be observed using IHC methods, H&E staining, and a light microscope [31]. Testicular tissue repair was confirmed by the regeneration of the seminiferous tubules, based on observing of seminiferous tubules become intact and compact again [27].

In this study, light microscopy examination revealed that testicular tissue repair occurred in the T2 group. The overview of the testicular tissue repair can be compared with that in the positive (fertile) control group, which did not experience testicular degeneration and remained in the normal condition (Figure-4 and Table-2), whereas the T1 group did not exhibit improvement in their testicular tissue. The tissue damage was comparable with that in the negative (infertile) control group with testicular degeneration (Figure-4 and Table-2).

## Conclusion

Transplantation of MSCs cultured under hypoxic conditions is an effective treatment for testicular failure in a rat infertility model.

## Authors’ Contributions

ES: Supervised the whole study. ES and HP: Designed the study, analyzed the data, wrote and edited the article. Both authors read and approved the final manuscript.

## References

[1] Safitri E, Utama S, Bumi C, Mulyani S.W.M (2014). Hypoxic preconditioning for viable and self-renewing mesenchymal stem cells (MSCs) as the regeneration of spermatogenesis process. Adv. Nat. Appl. Sci.

[2] Safitri E, Hariadi M (2019). Comparison of biotechnological culture of hypoxia-conditioned rat mesenchymal stem cells with conventional *in vitro* culture of normoxia-conditioned rat mesenchymal stem cells for testicular failure therapy with low libido in rats. Vet. World.

[3] Cakici C, Buyrukcu B, Duruksu G (2013). Recovery of fertility in azoospermia rats after injection of adipose-tissue-derived mesenchymal stem cells:Tha sperm generation. Bio. Med. Res. Inter.

[4] Safitri E, Srianto P, Widiyatno T.V, Sandhika W, Prasetyo R.H (2018). Viability of rabbit adipocyte stem cells cultured under different oxygen concentrations *in vitro*. Philippine J. Vet. Med.

[5] Li L, Chen X, Wang W.E, Zeng C (2016). Review article:How to improve the survival of transplanted mesenchymal stem cell in ischemic heart?. Stem. Cells Inter.

[6] Scaradavou A, Smith K.M, Hawke R, Schaible A, Abboud M, Keman N.A, Young J.W, Barker J.N (2015). Cord blood units with low CD34+cell viability have a low probability of engraftment after double unit transplantation. Biol. Blood Marrow Transplant.

[7] Tsai C.C, Chen Y.J, Yew T.L, Chan L.L, Wang J.Y, Chiu C.H, Hung S.C (2011). Hypoxia inhibits senescence and maintains mesenchymal stem cell properties through down-regulation of E2A-p21 by HIF-TWIST. Blood J. Hematol.

[8] Mulyani S.W.M, Setiawati E.M, Safitri E, Astuti E.R (2014). The role of heat shock protein 27(HSP 27) as inhibitor apoptosis in hypoxic conditions of bone marrow stem cell culture. Dent. J. Maj. Ked. Gigi.

[9] Szablowska-Gadomska I, Zayat V, Buzanska L (2017). Influence of low oxygen tension on expression of pluripotency genes en stem cells. Acta Neuroiol. Exp.

[10] Cai B, Li X, Wang L.Y, Liu Y, Yang F, Chen H, Yin K, Tan X, Tan X, Zhu J, Pan Z, Wang B, Lu Y (2013). Apoptosis of bone marrow mesenchymal stem cells caused by homocysteine via activating JNK signal. PLoS One.

[11] Chen T.L, Zhu G.L, Wang J.A, Wang Y, He X.L, Jiang J (2014). Apoptosis of bone marrow mesenchymal stem cells caused by hypoxia/reoxygenation via multiple pathways. Int. J. Clin Exp. Med.

[12] Teixeira F.G, Panchalingam K.M, Anjo S.I, Manadas B, Pereira R, Sousa N, Salgado A.J, Behie L.A (2015). Do hypoxia/normoxia culturing conditions change the neuroregulatory profile of Wharton Jelly mesenchymal stem cell secretome?. Stem Cell Res. Ther.

[13] Qin H.H, Filippi C, Sun S, Lehec S, Dhawan A, Hughes R.D (2015). Hypoxic preconditioning potentiates the trophic effects of mesenchymal stem cells on co-cultured human primary hepatocytes. Stem Cell Res. Ther.

[14] Zhou H, Li D, Shi C, Xin C (2015). Effects of exendin-4 on bone marrow mesenchymal stem cell proliferation, migration and apoptosis *in vitro*. Sci. Rep.

[15] Galleu A, Riffo-Vasquez Y, Trento C, Lomas C, Dolcetti L, Cheung T.S, Bono N.V, Barbiene R, Halai K, Ward S, Weng L, Chikraverty R, Lombardy B, Watt F.M, Orchad K, Marks D.J, Apperly J, Bornhouser M, Welezak H, Bennet C, Dazzi F (2017). Apoptosis in mesenchymal stromal cells induces *in vivo* recipient-mediated immunomodulation. Sci. Transl. Med.

[16] Suda T, Takubo K, Semenza G.L (2011). Cell stem cell review. Metabolic regulation of hematopoietic stem cells in hypoxic niche. Cell Stem Cell.

[17] Nakamura-Ishizu A, Takizawa H, Suda T (2014). Reviews:The analysis, roles and regulation of quiescence in hematopoietic stem cells. Development.

[18] Takubo K (2011). The hypoxia regulatory system in hematopoietic stem cells. Adv. Hemato. Stem Cell Res.

[19] Yue F, Bi1 P, Wang C, Shan T, Nie W, Ratliff T, Gavin T.P (2017). Pten is necessary for the quiescence and maintenance of adult muscle stem cells. Nat Comm.

[20] Cheung T.H, Rando T.A (2013). Molecular regulation of stem cell quiescence. Nat. Rev. Mol. Cell Biol.

[21] Rumman M, Dhawan J, Kassem M (2015). Concise review:Quiescence in adult stem cells:Biological significance and relevance to tissue regeneration. Stem. Cells.

[22] Daignan-Fornier B, Sagot I (2011). Proliferation/quiescence:When to start?Where to stop?What to stock?. Cell Div.

[23] Kolf C.M, Cho E, Tuan R.S (2007). Review mesenchymal stromal. Biology of adult mesenchymal stem cells:Regulation of niche, self-renewal, and differentiation. Arthritis Res. Ther.

[24] Kang X.Q, Zang W.J, Song T.S, Xu X.L, Yu X.J, Li D.L, Meng K.W, Wu S.L, Zhao Z.Y (2005). Rat bone marrow mesenchymal stem cells differentiate into hepatocytes *in vitro*. World J. Gastroenterol.

[25] Gala K, Burdzińska A, Idziak M, Makula J, Paczek L (2011). Characterization of bone-marrow-derived rat mesenchymal stem cells depending on donor age. Cell Biol. Int.

[26] Eleotério R.B, Sepúlveda R.V, Reis E.C.C, Falente F.L, Borges A.P.B (2016). Isolation, expansion and differentiation of mesenchymal stromal cells from rabbits'bone marrow. Pesq. Vet. Bras.

[27] Safitri E, Utama S, Widiyatno T.V, Sandhika W, Prasetyo R.H (2016). Auto-regeneration of mice testicle seminiferous tubules due to malnutrition based on stem cells mobilization using honey. Asian Pac. J. Reprod.

[28] Hackbert L, Heiman J.R (2002). Acute dehydroepiandrosterone (DHEA) effect on sexual arousal in postmenopausal women. J. Women Health Gend. Based Med.

[29] Hafez E.S.E, Hafez B (2010). Reproduction in Farm Animals.

[30] França L.R, Ogawa T, Avarbock M.R, Brinstel R.L, Russell L.D (1998). Germ cell genotype controls cell cycle during spermatogenesis in the rat. Biol. Reprod.

[31] Razi M, Najafi G, Feyzi S, Karimi A, Shahmohammadloo S, Nejati V (2012). Histological and histochemical effects of glyphosate on testicular tissue and function. Iran. J. Reprod. Med.

[32] Nowak M, Madej J, Dziegiel P (2007). Intensity of COX2 expression in cells of soft tissue fibrosarcomas in dogs as related to grade of tumour malignancy. Bull Vet. Inst. Pulawy.

[33] Widjiati W, Rachmawati A, Sosiawati S.M (2012). Identification of epidermal growth factor (EGF) protein in 46 kDa from oocyte maturation *in vitr*o of cow with. J. Vet. Med.

[34] Nabavi N, Todehdehghan F, Shiravi A (2013). Effect of caffeine on motility and vitality of sperm and *in vitro* fertilization of outbreed mouse in T6 and M16 media. Iran J. Reprod. Med.

[35] Widjiati W, Pusporini S.E, Arifin M.Z (2012). Comparative of fertility rate and barriers to development of mice embryos that cultured between M16 medium and human tubal fluid. J Vet Med,.

[36] Jin H, Shu Y, Dai S (2014). The value of second polar body detection 4 hours after insemination and early rescue ICSI in preventing complete fertilisation failure in patients with borderline semen. Reprod. Fertil. Dev.

[37] Youssef A, Han V.K.M (2016). Low oxygen tension modulates the insulin-like growth factor-1 or -2 signaling via both insulin-like growth factor-1 receptor and insulin receptor to maintain stem cell identity in placental mesenchymal stem cells. Endocrinology.

[38] Takubo K, Suda T (2012). Roles of the hypoxia response system in hematopoietic and leukemic stem cells. Int. J. Hematol.

[39] Forristal C.E, Wright K.L, Hanley N.A, Oreffo R.O.C, Houghton F.D (2010). Hypoxia inducible factor regulate pluripotency and proliferation in human embryonic stem cells cultured at reduced oxygen tensions. Reproduction.

[40] Li L, Bhatia R (2011). Stem cell quiescence. Clin. Cancer Res.

[41] Arai F, Suda T (2008). Quiescent Stem Cells in the Niche. The Stem Cell Research Community, StemBook, London.

[42] Grskovic M, Santos M.R (2008). The Pluripotent Transcriptome. The Stem Cell Research Community, StemBook, London.

[43] Yamanaka S (2007). Strategies and new developments in the generation of patient-specific pluripotent stem cells. Stem Cell Rev.

[44] Lavagnolli T, Gupta P, Hormanseder E, Botenbal H.M, Dharmalenghan G, Carroll T, Gurdon J.B, Fisher A.G, Markensclanger M (2014). Initiation and maintenance of pluripotency gene expression in the absence of cohesion. Genes Dev.

[45] Festuccia N, Osorno R, Wilson V, Chambers I (2013). The role of pluripotency gene regulatory network components in mediating transitions between pluripotent cell states. Curr. Opin. Genet, Dev.

[46] Dengler V.L, Galbraitmh M, Espinosa J.M (2014). Transcriptional regulation by hypoxia ınducible factors. Crit. Rev. Biochem. Mol. Biol.

